# Editorial: Advances in Dietary Fat Absorption

**DOI:** 10.3389/fphys.2021.707403

**Published:** 2021-06-08

**Authors:** Andromeda M. Nauli, Sylvia Santosa, J. Brandon Dixon

**Affiliations:** ^1^Department of Pharmaceutical Sciences, College of Pharmacy, Marshall B. Ketchum University, Fullerton, CA, United States; ^2^Department of Health, Kinesiology, and Applied Physiology, Concordia University, Montreal, QC, Canada; ^3^George W. Woodruff School of Mechanical Engineering, Parker H. Petit Institute for Bioengineering and Bioscience, Georgia Institute of Technology, Atlanta, GA, United States

**Keywords:** lipid, intestine, chylomicron, VLDL, lymph, bile acid, apolipoprotein, lipoprotein

Cardiovascular disease (CVD) remains as the leading cause of death in the world (WHO, 2020). One of the main risk factors of CVD is dyslipidemia. Treatment of dyslipidemia through pharmacological or lifestyle interventions has been shown to decrease cardiovascular-related mortality (Shalev et al., 2009; Haglin et al., 2011), indicating the importance of targeting dyslipidemia in the prevention and treatment of CVD. Dyslipidemia is caused by dysregulation of lipoprotein metabolism, a significant component of which is dietary fat absorption. Therefore, it is essential to understand how the small intestine absorbs dietary lipids and transport them as lipoproteins, namely chylomicrons and very low-density lipoproteins (VLDLs).

Cifarelli et al. recently led a Research Topic that addresses the importance of the lymphatic system in lipid metabolism (Cifarelli et al., 2021). Our topic “*Advances in Dietary Fat Absorption”* focuses on how the small intestine regulates dietary fat absorption and the production of chylomicrons and VLDLs. We have curated several research articles that speak to this important issue.

In the Review article, Levy et al. discuss congenital defects of dietary fat absorption and how these defects shape our understanding of chylomicron and intestinal VLDL biogenesis. Some of the genetic defects discussed include MTTP gene mutations that lead to abetalipoproteinemia, APOB gene alterations that lead to familial hypobetalipoproteinemia, SAR1B gene defects that are associated with chylomicron retention disease, and CD36 deficiency that is linked to increased plasma triglycerides. Our understanding of chylomicron and intestinal VLDL biogenesis has contributed to the development of several important lipid-lowering drugs, such as MTP inhibitor (lomitapide) and cholesterol absorption inhibitor (ezetimibe). NPC1L1, the target of ezetimibe, is important not only for cholesterol uptake but possibly also for cholesterol esterification and intracellular trafficking (Nakano et al., 2020).

The cholesterol-derived bile acid, which is arguably one of the most important physiological fat emulsifiers, is critical for dietary fat absorption. Drugs that sequester bile acids can be used to lower cholesterol and are safe for pregnant and nursing women (Kusters et al., 2010). In inflammatory bowel disease, particularly Crohn's disease, there is a bile acid malabsorption. In the Mini Review article, Fitzpatrick and Jenabzadeh discuss this malabsorption from both the pre-clinical and clinical aspects. Importantly, Crohn's disease patients with severe bile acid malabsorption display reduced intraduodenal bile acid and impaired micelle formation. Even though bile acid malabsorption is common in these patients, it has not been routinely assessed in clinical practice (Vitek, 2015).

As mentioned above, the atherogenic lipoproteins that are produced by the small intestine are chylomicrons and VLDLs. Unlike the hepatic lipoproteins that contain ApoB100, the intestinal atherogenic lipoproteins contain ApoB48. One of the reasons that the small intestine produces ApoB48 instead of ApoB100 has previously been provided by Lo et al. Relative to ApoB100, ApoB48 could mediate more dietary lipid transport from the intestine to the lymph when challenged with a moderate dose (6 μmoles/h) of triglycerides (Lo et al., 2008). However, when challenged with a high dose (8 μmoles/h) of triglycerides, Lo and Coschigano report in the Perspective article that the transport of dietary lipids to the lymphatic circulation was comparable between the ApoB48-producing intestine and the ApoB100-producing intestine. Therefore, their studies collectively suggest that the higher efficiency of ApoB48 relative to ApoB100 in mediating dietary lipid transport from the intestine seemed to disappear when a higher dose of triglycerides was given.

The Original Research article by Trevaskis et al. provides novel findings that convey that intestinal lymphatic flow, lipid transport, and drug transport in larger animals, such as dogs, are more similar to humans. This is particularly salient as studies performed in small animals, such as mice, have a tendency to underestimate the lymphatic drug transport in humans. Based on their findings, the authors also suggest that lymphatic drug transport data collected from larger animal models can be used to predict lymphatic drug transport in humans *via* allometric scaling.

In the Brief Research Report, McMillan et al. show that patients with spinal cord injury displayed a smaller peak in the increase of dietary triglycerides in their blood. Although the difference in amplitude was not statistically significant, the level of spinal cord injury correlated strongly with time-to-peak dietary triglyceride in the blood. In other words, the more superior the location of the injured spinal cord was, the slower the dietary triglycerides reached their peak in the blood. Their studies suggest that spinal cord damage could possibly disrupt dietary fat absorption.

Although the basic physiological processes of dietary fat absorption are well understood, there are several remaining challenges that need to be addressed. From the perspective of cell and molecular biology, more studies need to be performed to get a better understanding of the mechanisms of chylomicron packaging and release by the enterocytes. From the perspective of integrative physiology, it is important to delineate further how dietary fat absorption contributes to the progression of dyslipidemia. As indicated above, spinal cord injury may affect both dietary fat absorption and lipoprotein metabolism, but their interplay needs to be elucidated. Different sexes also tend to be different in their dietary fat absorption, body fat deposition, and lipoprotein metabolism (Nauli and Matin, 2019). All of these complex relationships warrant further investigation in order to make our understanding of these physiological processes less compartmentalized.

## Author Contributions

AN wrote the manuscript. SS provided feedback to the manuscript. AN, SS, and JD served as the topic guest editors. All authors contributed to the article and approved the submitted version.

## Conflict of Interest

The authors declare that the research was conducted in the absence of any commercial or financial relationships that could be construed as a potential conflict of interest.
